# Exploring the Role of Lower Genital Tract Microbiota and Cervical–Endometrial Immune Metabolome in Unknown Genesis of Recurrent Pregnancy Loss

**DOI:** 10.3390/ijms26031326

**Published:** 2025-02-04

**Authors:** Sergey A. Mikhalev, Mark A. Kurtser, Victor E. Radzinsky, Mekan R. Orazov, Narasimha M. Beeraka, Lyudmila M. Mikhaleva

**Affiliations:** 1 Federal State Autonomous Educational Institution of Higher Education “N.I. Pirogov Russian National Research Medical University” of the Ministry of Health of the Russian Federation, 117997 Moscow, Russia; mikhalev@me.com (S.A.M.); m.kurtser@mcclinics.ru (M.A.K.); 2 City Clinical Hospital No. 31 Named After Academician G.M. Savelyeva of the Department of Health, 119415 Moscow, Russia; 3 Department of Obstetrics and Gynecology, Federal State Autonomous Educational Institution of Higher Education «Peoples’ Friendship University of Russia», 117198 Moscow, Russia; radzinsky@mail.ru (V.E.R.); omekan@mail.ru (M.R.O.); 4 Department of Human Anatomy and Histology, I.M. Sechenov First Moscow State Medical University of the Ministry of Health of the Russian Federation (Sechenov University), 119991 Moscow, Russia; 5 Raghavendra Institute of Pharmaceutical Education and Research (RIPER), Chiyyedu 515721, Andhra Pradesh, India; 6 Department of Studies in Molecular Biology, University of Mysore, Mysore 570006, Karnataka, India; 7 Scientific Research Institute of Human Morphology Named After Academician A.P. Avtsyn of the Federal State Budgetary Scientific Institution “Russian Scientific Center of Surgery Named After Academician B.V. Petrovsky”, 125315 Moscow, Russia

**Keywords:** microbiota, recurrent pregnancy loss, miRNAa, HPV, immunity, p16/Ki-67 markers

## Abstract

Recurrent pregnancy loss (RPL) of unknown genesis is a complex condition with multifactorial origins, including genetic, hormonal, and immunological factors. However, the specific mechanisms underlying endocervical cell proliferation disorders in women with RPL remain inadequately understood, particularly concerning the role of microbiota and viral infections. The aim of this study was to investigate the mechanisms of endocervical cell proliferation disorders in women with RPL of unknown genesis by examining microbiota, human papillomavirus (HPV) typing, and the expression levels of key molecular biological markers, including p16/Ki-67, BCL-2, miR-145, and miR-34a. A prospective observational comparative study was executed on women with RPL and healthy pregnant controls with full ethical approval. Samples were collected for HPV typing and immunocytochemical analysis to evaluate the expression of p16, Ki-67, BCL-2, and the anti-oncogenic microRNAs (miR-145 and miR-34a). The expression of mRNA for the progesterone receptor (PGR-A) was also assessed, alongside local immune status markers, including proinflammatory T-lymphocytes (Th17/Th1) and regulatory CD4+ Tregs. Overexpression of p16, Ki-67, and BCL-2 was observed in 52.5% of women with RPL who had an ASC-US/LSIL cytogram, with the average double expression of p16/Ki-67 being three times higher than in the healthy pregnant group. A significant decrease in PGR-A mRNA expression in the endocervix of women with RPL was noted, accompanied by a dysregulated local immune status characterized by an increased prevalence of Th17/Th1 cells and a reduction in regulatory CD4+ Tregs. Additionally, the expression of miR-145 and miR-34a in the endocervix and endometrium of women with RPL significantly differed from the physiological pregnancy group, particularly in the context of high-risk HPV infection. The findings describe that disorders of endocervical cell proliferation in women with RPL of unknown genesis are associated with overexpression of specific molecular markers, impaired immune regulation, and altered microRNA profiles. These alterations may contribute to the pathophysiology of RPL, highlighting the need for further research into targeted interventions that could improve reproductive outcomes in affected individuals.

## 1. Introduction

Recurrent pregnancy loss (RPL), with a rising global incidence, affects approximately 1 in 100 pregnancies and remains a multifaceted public health issue lacking a universally accepted resolution [1]. RPL is defined by the occurrence of two or three spontaneous miscarriages before the 24th week of pregnancy. The mechanisms underlying RPL are not well understood [2]. It is considered a polyetiological condition influenced by numerous modifiable and non-modifiable factors, including immunological, hormonal, genetic, environmental, hematological factors, and the reproductive tract microbiome. However, the genesis of nearly half of these cases remains unexplained [3,4]. Recent studies have identified the activation of opportunistic and pathogenic microorganisms as detrimental to pregnancy outcomes, often associated with hormonal imbalances and physiological immunosuppression. The cervical and vaginal microecology reflects the interactions between microbial communities and the host, with the pH (3.8 to 4.5) maintained through microbial and cervicovaginal epithelial lactic acid generation, influenced by progesterone levels [5].

The cervical and vaginal microbial communities are dynamic ecosystems that undergo typical alterations at the time of pregnancy [6]. These alterations, driven by microbial competition, and other factors including hormonal, immunological, and metabolic interactions can impact reproductive outcomes [7]. The dominance of *Lactobacilli* in cervicovaginal microbiota of the majority of the women is seen as an indicator of a stable environment conducive to full-term delivery, particularly in the second trimester, regardless of initial parameters [8]. Establishing a direct causal link between cervical microbiota and spontaneous preterm birth (PTB) is challenging. However, advancements in sequencing technologies have facilitated the identification of microbial markers that are potentially harmful to pregnancy. The role of cervicovaginal microbiota in PTB is supported by associations with the proliferation of opportunistic microorganisms in conditions such as bacterial vaginosis (BV) and aerobic vaginosis, involving species like *Gardnerella*, *Atopobium*, *Prevotella*, *Peptostreptococcus*, *Mobiluncus*, *Sneathia*, *Leptotrichia*, *Megasphaera* spp., and *Mycoplasma* [9,10,11].

The association between HPV and vaginal dysbiosis has been demonstrated in the studies of many authors, especially when highly oncogenic genotypes are identified. Three meta-analyses have confirmed correlations between non-lactobacilli-dominated vaginal microbiota and cervical disease through the influence of the microbiome on the probability of HPV infection, persistence, and the development of precancerous changes [12,13]. The relationship between HPV infection and pregnancy outcomes is unclear, as are potential mechanisms, either directly or immune-mediated, that provoke RPL [14]. When analyzing the HPV status (positive (carrier status without cervical diseases; HPV-associated cervical diseases), negative and after vaccination) on pregnancy outcomes (term spontaneous labor: n = 4942; preterm birth (PTB): n = 386; spontaneous miscarriage: n = 270) in a large-scale study, an adverse effect was found only in the case of morphological changes in the cervix [15]. The role of HPV infection in the development of pregnancy complications (spontaneous abortion, PTB, placental abnormalities, fetal growth retardation, premature rupture of membranes (PROM), preeclampsia) is confirmed by the results of morphological studies of the placenta [16]. A group of researchers, when studying samples of decidual tissue and chorionic villi of 118 patients with spontaneous miscarriage, detected HPV in 65.0% [17]. In a major review of 36 studies, Niyibizi et al. showed a significant role of persistent viral infection in the development of adverse reproductive outcomes (PTB) (stillbirth, fetal growth restriction), but without increasing the risk of RPL [18]. The cervicovaginal microbiota is affected by the innate immune factors [19]. HPV infection and its persistence may be supported by altered immune adaptations that promote immune tolerance toward semi-allogeneic fetal tissues. This immunological shift could enable HPV to evade immune detection and clearance, fostering a microenvironment that allows the virus to persist [20]. Modern cervical screening strategies, including testing with HPV genotyping and cytology, are linked to a greater risk of unjustified colposcopies and biopsies that occur when HPV-positive results are detected [21].

The double staining method with the study of molecular biomarkers p16/Ki-67 is considered as a promising marker of oncogenic transformation mediated by infection with high-risk (HR) HPV in cervical cancer screening [21]. p16INK4a (p16) is a protein, an inhibitor of cyclin-dependent kinase, implicated in the regulation of cell cycle (mitosis). Inactivation of the retinoblastoma mitotic division suppressor gene (pRb) mediated by viral mRNA E6/E7 leads to a significant increase in the p16 level in cells, which indicates a violation of the regulation of cell division. Overexpression of p16 in precancerous changes and cervical cancer has been studied fairly extensively [22,23]. The interaction of E6/E7 with the tumor suppressor protein p53 and retinoblastoma protein (pRb) leads to a decrease in cellular apoptosis, activation of pathological proliferation, neoangiogenesis, and tumor transformation [24]. The regulation of cellular immune processes in the cervix is carried out by progesterone or its mediators (progesterone-induced blocking factor, PIBF), which contribute to the maintenance of immune homeostasis due to the differentiation of regulatory T cells (Tregs), both systemically and at the mother–fetus border (decidual membrane and placenta) [25,26,27].

The probability of RPL development due to aberrant DNA methylation of imprinted, placenta-specific, and immune-related genes is poorly understood [28]. MicroRNAs (miRNAs, miRs) are important components of post-transcriptional gene regulation and provide control over the expression of various genes by pairing with a complementary sequence based on mRNA molecules and inhibiting their translation [29,30]. MicroRNAs play a crucial role in various physical processes of the cell, and their dysregulation is the cause of the development of many diseases [31,32]. Considering gene mutations can provide stimulation of microRNA-mediated regulation of cellular functions and lead to certain disorders in a number of biological processes, including embryo implantation, placental development, and the interaction between the immune sequences of mother and fetus [33,34]. The relationship between abnormal miR expression and various human malignancies, including cervical cancer, has been shown. MicroRNAs have been reported to regulate the expression of genes involved in the formation of the “implantation window” and endometrial maturation disorders, but these results are contradictory. Endometrial and circulating miRNAs have been proposed as potentially reliable biomarkers of implantation and repeated implantation losses as well as miscarriage [35,36,37]. The expression of miR-145, miR-155-5p, miR-20b-3p, and miR-330-5p in women with repeated implantation failures was reduced [38]. Furthermore, research suggests that immune-metabolic interactions in the cervix and endometrium play crucial roles in maintaining pregnancy. Dysregulation in these interactions may contribute to RPL by affecting the immune tolerance and metabolic environment essential for embryo implantation and growth. Understanding the interplay between the immune system, metabolic pathways, and microbial communities offers new avenues for diagnosing and potentially treating RPL. As our knowledge of these complex interactions grows, so does the potential for developing targeted therapies that could mitigate the risk of RPL and improve reproductive outcomes for affected individuals. The unclear role of cervicovaginal microbiota disorders, viral infections (such as HR HPV), alterations in local immunity, and molecular biological changes in the endocervix in the genesis of RPL impedes the development of personalized prevention and treatment strategies. The objective of this research is to evaluate the expression of molecular biological factors in the endocervix (p16, Ki-67, Th17, Th1, CD4+ Treg, BCL-2, PGR-A) and endometrium (miR-145, miR-34a) of women with RPL of unknown genesis compared to the control group.

## 2. Results

The average age of women with RPL was 27.3, ranging from 18 to 40. For the group of healthy pregnant women, the indicator was slightly higher, but the differences were not statistically significant (Table 1).

The average BMI values of women in both groups, as well as obesity rates, were comparable. No significant differences in the incidence of other diseases, such as urinary tract infections, hypertension, diabetes mellitus and anemia, were found in the groups. The frequency of anemia and obesity in the groups with RPL and the control group did not influence the conclusion. The characteristics of the vaginal microbiota of women with RPL as well as healthy pregnant women are presented in Table 2.

When studying the types of microbiota, vaginal normocenosis was detected significantly more often in patients with successful pregnancy outcomes than with RPL (*p* < 0.001). Microbiota disorders prevailed in the main group (81.6%) compared to healthy pregnant women (42.4%), with a higher frequency of candida vaginitis and bacterial vaginosis. The proportion of women with normal flora (Nugent scores 0–3) in the group with RPL was significantly lower than in healthy pregnant women (18.8% versus 42.9%, *p* = 0.004, χ^2^ = 8.8). Smear samples with intermediate flora (4–6 points on the Nugent scale) and bacterial vaginosis (7–10 points) were more common in the group with RPL (43.0% and 38.3%, respectively) compared to healthy pregnant women (37.1% and 20.0%, respectively), but the differences were not statistically significant. The frequency of BV due to the predominance of Gardnerella vaginalis was significantly higher in the group with RPL (29.7% versus 6.8%, *p* < 0.001). No differences in the frequency of nonspecific vaginitis (Gram-positive cocci and Gram-negative rods) were found in the groups.

The results of vaginal discharge culture showed a spectrum of different microorganisms with a sample with a high diagnostic titer (105 degree) (Table 3). In the group with RPL, *Enterococcus* spp. and *Streptococcus agalactiae* cultures in vaginal discharge were detected with a higher frequency and in high diagnostic titers (more than 105–107) compared to healthy pregnant women. The frequency of other microorganisms such as *Corynebacterium* spp., *Klebsiella* spp., *Staphylococcus epidermidis*, *Corynebacterium* spp., *Actinomyces* spp., *Bacillus* spp., *Staphylococcus* spp. and *Streptococcus* spp. in the groups was comparable and did not show a correlation with RPL.

The revealed inflammatory disorders of the vaginal microbiota in women with recurrent pregnancy loss (RPL) have become the basis for the use of antimicrobial drugs and biocenosis restoration.

**Figure 1:**
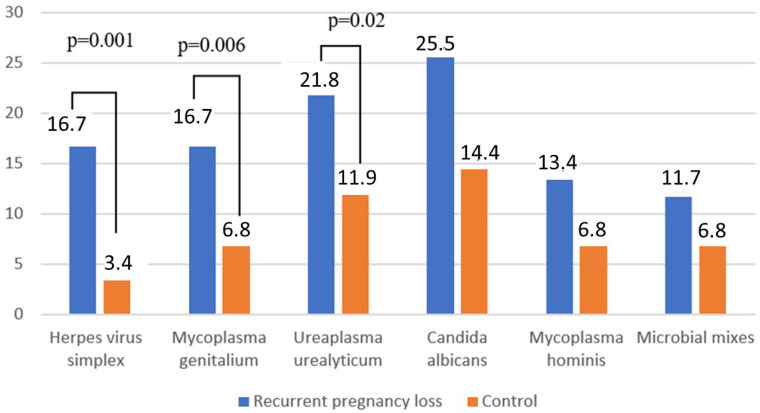
PCR analysis of cervical discharge from women with recurrent pregnancy loss (RPL) and healthy pregnant women. This comparison reveals intergroup differences in pathogen prevalence, with statistically significant results indicating potential disparities in cervical microbial environments associated with RPL. *p* = 0.001, *p* = 0.006, *p* = 0.02 < 0.05 signifies statistically significant intergroup differences.

When testing for STIs, no specific pathogens were detected in either group, but HSV2 was detected more frequently in women with RPL than in healthy pregnant women (*p* = 0.001, χ^2^ = 13.0) (Figure 1).

In the RPL group, a significantly higher level of Mycoplasma genitalium (*p* = 0.006, χ^2^ = 7.6) and *Ureaplasma urealyticum* (*p* = 0.02, χ^2^ = 5.1) infections were detected compared to healthy pregnant women. In the RPL group, Mycoplasma hominis was noted more often than in the control group, but no statistically significant differences were found. Microbial mixes were detected in 9.2% of women and *Candida albicans* was revealed with comparable frequency. The combination of ureaplasma infection with BV was significantly more common in the RPL group (75.0%) compared to healthy pregnant women (21.4%).

The frequency of HPV detection in RPL (n = 113) was three times higher than in the control group (n = 19) (47.3% versus (16.1%) (*p* < 0.001; χ^2^ = 40.3) (Table 4).

HPV 16 was the most common genotype among HPV-positive samples and was more common in the RPL group than in the control group (*p* = 0.001; χ^2^ = 16.8). High HPV HR viral load (more than 105 HPV DNA in 1 mL) was detected in 68 women with RPL (60.2%) versus 2 (22.2%) in the control group (*p* = 0.005; χ^2^ = 8.1). Vaginal biotope violations caused by the prevalence of vaginosis-associated bacteria were detected in 85 (75.2%) women with HPV HR. A combination of two or more HPV types was found in 68 (60.2%) women with HPV HR.

Variants of cytological conclusions on the state of the exo- and endocervix in accordance with the classification according to the Bethesda system (2001) of women with RPL and healthy pregnant women are presented in Table 5.

In most women in both groups, the test results corresponded to the cytological norm (NILM), the frequency of abnormal cytograms (ASC-US, LSIL) in the group with RPL was higher (37.3%) than in the control group (19.5%).

Cervical epithelial samples were used to assess the expression of mRNA of genes regulating cell proliferation and the mitotic cycle (p16, Ki-67), PGR-A and BCL-2, which has an anti-apoptotic effect (Table 6).

The cytology results with double staining of p16/Ki-67 were statistically more often noted in the group with RPL compared to healthy pregnant women (*p* < 0.001, χ^2^ = 7.9).The increased expression of markers of impaired cell proliferation was observed not only in 52.3% of patients with miscarriage with a normal colposcopic picture, but also in 18.2% of healthy women with successful reproductive outcomes.

**Figure 2:**
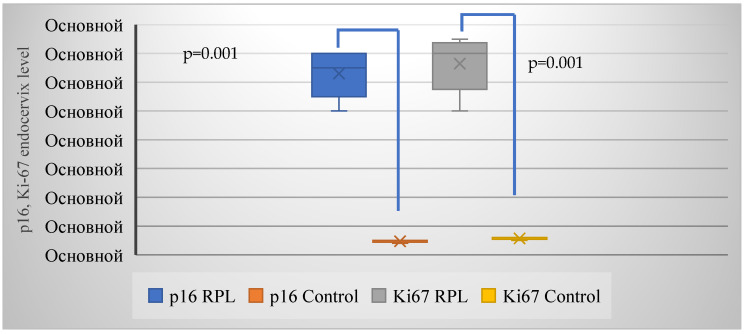
Comparative expression analysis of p16 and Ki-67 markers in the endocervix of women with RPL versus healthy pregnant women. Elevated p16 and Ki-67 expression levels suggest altered cellular proliferation and regulatory dynamics in the endocervical epithelium of women experiencing RPL, with *p* = 0.001 < 0.05 denoting statistically significant differences. Note: Ocнoвнoй: Basic.

The average expression of p16/Ki-67 in the group with RPL was 0.65 and 0.7, respectively, and 0.05 and 0.06 in the control group, respectively (Figure 2).

It was revealed that all samples with positive double staining of p16/Ki-67 were HPV HR-positive. The expression of BCL-2 significantly prevailed in the RPL group; in half of the women, it was significantly higher than in healthy pregnant women (*p* = 0.002, χ^2^ = 10.1).

Impaired receptor activity of PGR-A in endocervical cells was detected exclusively in women with RPL (50.0%), which allows the marker to be included in a complex of studies in women with RPL of unknown genesis.

**Figure 3:**
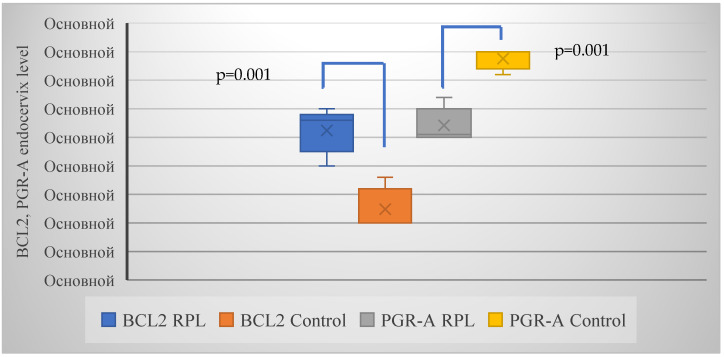
Frequency of overexpression of BCL-2 and hypoexpression of PGR-A in the endocervix of women with RPL compared to a group of healthy pregnant women. *p* = 0.001, which is <0.05 denotes statistically significant intergroup differences. Note: Ocнoвнoй: Basic.

The average expression of BCL-2 and PGR-A in the group with RPL was 0.28 and 0.25, respectively, and 0.1 and 0.4 in the control group, respectively (Figure 3).

A decrease in the expression of mRNA of progesterone receptors PGR-A indicates a change in the cellular structure of the endocervix under the influence of persistent HPV infection. This category of patients, with a decrease in the reproduction of progesterone receptors and a decrease in receptor sensitivity, was distinguished by resistance to progesterone drugs. The results of the analysis of the immunoregulatory index of immunocompetent cells of the endocervix of women with RPL and the control group are presented in Table 7.

Flow cytometry revealed a pronounced imbalance in the Th17/Th1 ratio in the endocervical contents of patients with RPL, indicating the predominance of the proinflammatory immune response. The index of regulatory T-lymphocytes in the endocervix of healthy pregnant women was 72.7% and 22.7% in the group with RPL.

A high index of Th17/Th1 lymphocytes in the endocervix with a decrease in the expression of protective CD4+ T-lymphocytes in the group of women with RPL may indicate immunoregulatory disorders of tolerance to the semi-allogenic fetus. With a shift in lymphocytes towards Th17/Th1 and a decrease in fetus-tolerant CD4+ Tregs, there is a risk of pregnancy termination.

**Figure 4:**
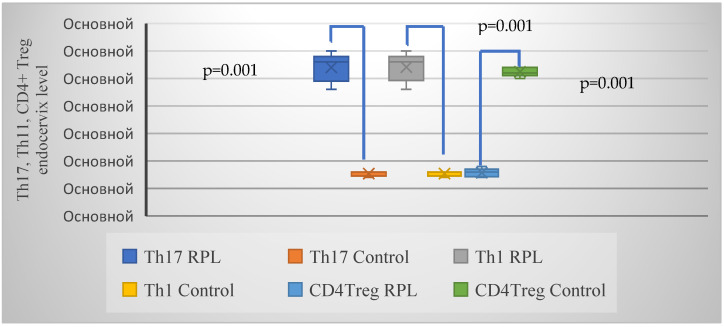
Frequency of overexpression of markers Th17, Th1 and hypoexpression of CD4+ Treg in the endocervix of women with RPL and healthy pregnant women. This profile assesses immunological markers potentially linked to cervical immune tolerance or inflammatory responses in RPL. *p* = 0.001, which is <0.05 indicates statistically significant intergroup differences. Note: Ocнoвнoй: Basic.

The average expression of Th17, Th1 and CD4+ Treg in the group with RPL was 2.8, 2.8 and 0.8, respectively, and 0.8, 0.8 and 2.6 in the control group, respectively (Figure 4).

With a shift in lymphocytes towards Th17/Th1 and a decrease in fetus-tolerant CD4+ Tregs, there is a risk of pregnancy termination. RT-PCR with reverse transcription was used to assess the expression level of miR-145 and miR-34a in the endocervix of the examined women (Table 8).

There is a significant decrease in the expression of miR-145 and miR-34a in the endocervix of patients with ASC-US and LSIL in the group with RPL, as well as in healthy pregnant women, which is a result of impaired cell proliferation.

**Figure 5:**
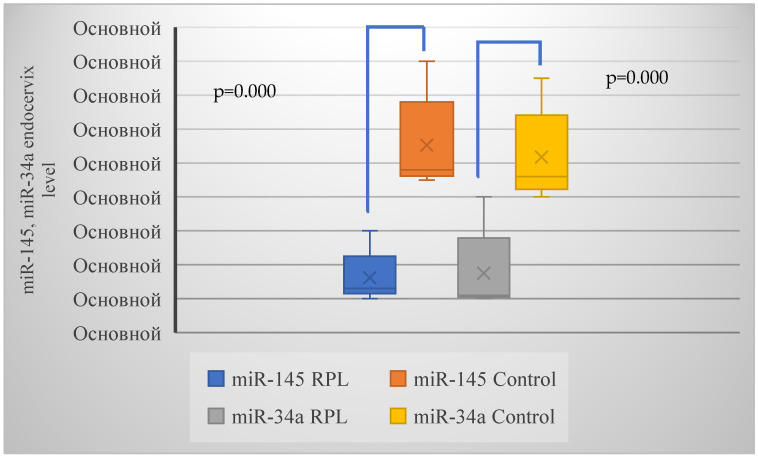
MicroRNA (miR) expression levels in the endocervix of women with RPL and healthy pregnant controls. Altered miR expression profiles suggest potential regulatory roles in cellular processes associated with RPL pathology. *p* = 0.000 < 0.05 denotes statistically significant intergroup differences. Note: Ocнoвнoй: Basic.

A decrease in the expression of anti-oncogenic miR-145 in the endocervix of women in the main group was significantly more common than in the control group (*p* = 0.002, χ^2^ = 10.1). Impaired expression of miR-34a also prevailed in the group with RPL—four times more often than in healthy pregnant women (*p* = 0.001, χ^2^ = 13.5). The average expression values of anti-oncogenic markers in the endocervix of women with RPL and healthy pregnant women are shown in Figure 5.

The average expression of miR-145 and miR-34a in the group with RPL was 0.13 and 0.11 respectively, and 0.48 and 0.46 in the control group, respectively.

The expression of miR-145 and miR-34a in the endometrium of women with RPL and healthy pregnant women depended on the presence of HPV of high oncogenic risk (HPV HR) (Table 9).

The expression of miR-145 and miR-34a in the endometrium of women with RPL and healthy pregnant women depended on the presence of HPV of high oncogenic risk (HPV HR). The expression of miR-145 and miR-34a in women with RPL and abnormal cytograms and HPV HR was significantly reduced in contrast to patients without viral infection (*p* < 0.05). Women with persistent HPV HR in the endocervix had statistically significantly lower expression of miR-145 and miR-34a in the endometrium than those without any viral infection (*p* < 0.05). The expression of anti-oncogenic markers in the endometrium of healthy women was the highest in comparison with patients with viral infection (*p* < 0.05).

## 3. Methods

### 3.1. Study Design and Population

A prospective observational comparative study was conducted with a sample of women (n = 359) at the Department of Pregnancy Pathology and Gynecology of the Perinatal Center, City Clinical Hospital named after Academician G.M. Savelyeva, Moscow Department of Health. Participants were divided into two groups: the first group (n = 239) consisted of women with a visually unchanged cervix and recurrent pregnancy loss (RPL) of unknown genesis, while the second group (n = 118) comprised healthy pregnant women without adverse reproductive outcomes.

### 3.2. Inclusion and Exclusion Criteria

Inclusion criteria for the RPL group included women aged 18–45 with a history of two or more miscarriages, unexplained RPL etiology, visually unchanged cervix during speculum examination and colposcopy, and voluntary informed consent. Exclusion criteria were morphologically altered cervix (cervicitis, scars, dysplasia, cysts), malignant neoplasms related to the reproductive system, ongoing pregnancy or lactation, severe somatic diseases, diabetes mellitus, primary and secondary immunodeficiency and history of cesarean section or myomectomy.

### 3.3. Immunocytochemical Examination

For an immunocytochemical study of the endocervix, 66 women were randomly selected from the entire sample of women: n = 44 from the group with RPL and 22 from the group of pregnant women without adverse reproductive outcomes.

### 3.4. Sample Collection and Clinical Assessment

Endometrial samples were obtained from women with RPL and a comparison group one month after medical termination of pregnancy at 5–6 weeks (n = 22). Clinical and anamnestic data were collected, along with obstetric and gynecological status, cytological examination, and HPV typing. Microbial identification was conducted using Gram-stained vaginal smears, vaginal culture results, and vaginal pH. Bacterial vaginosis (BV) diagnosis followed Nugent criteria (score 0–3: normocenosis, 4–6: intermediate type; 7–10: positive).

### 3.5. Cytological Examination

Cervical epithelium samples were collected using a Cervix brush and processed with BD SurePath liquid cytology transport medium (BD Diagnostics, Franklin Lakes, NJ, USA). Cytological assessment adhered to the Bethesda system (2014), identifying normal cytograms (NILM), ambiguous smears (ASC-US), and precancerous conditions (LSIL, HSIL). Note: NILM: (Negative for Intraepithelial Lesion or Malignancy)—A normal cytology result indicating no evidence of precancerous or cancerous changes. It may still report benign findings such as infections or reactive changes. ASC-US (Atypical squamous cells of undetermined significance)—Slight abnormalities in squamous cells that are not clearly precancerous. Further testing, such as HPV DNA testing, may be needed for risk assessment. LSIL (Low-Grade squamous intraepithelial lesion)—Mild cellular abnormalities often associated with transient HPV infection. LSIL typically represents early-stage changes that may resolve spontaneously. HSIL (High-Grade squamous intraepithelial lesion)—More significant abnormalities indicating a higher risk of progression to cervical cancer if untreated. HSIL suggests moderate to severe dysplasia (CIN2 or CIN3).

### 3.6. HPV Detection and Typing

HPV DNA detection and typing were performed using “Proba GS” reagent kits (“DNA-Technology”, Moscow, Russia). The method involved cell lysis with a chaotropic agent, nucleic acid sorption, washing, and DNA elution. Amplification and typing of HPV DNA were conducted using the “Quantum-21” HPV PCR method, with fluorescence measurements on a “DT-964” device. Sexually transmitted infections (STIs) such as *Trichomonas vaginalis*, *Chlamydia trachomatis*, *Neisseria gonorrhoeae*, and *Mycoplasma genitalium* were detected by PCR.

### 3.7. Immunocytochemical Testing

Exo- and endocervical smears underwent immunocytochemical testing for mRNA expression of p16, Ki-67, BCL-2, and PGR using RNeasy commercial kits (QIAGEN, Germany). The reverse transcription reaction used reagents from “DNA-Technology”, Russia. The expression level was determined by real-time PCR. Immunohistochemical staining used anti-CDKN2A/p16INK4a antibodies (1:100 dilution, Abcam, Cambridge, UK) and pre-diluted monoclonal Ki-67 antibodies (Thermo Scientific, Waltham, MA, USA), with amplification via a labeled streptavidin–biotin–peroxidase system (Leica Bond-Max). Immunostaining was visualized with diaminobenzidine and hydrogen peroxide, analyzed by light microscopy.

### 3.8. Colposcopic Examination

Colposcopy was performed using a Leisegang device (Germany) with 7-15-30-fold magnification. The cervical mucosa was examined untreated, and then with 3% acetic acid and 2% aqueous Lugol’s solution (Schiller’s test). Targeted biopsy sites were identified using the Unified International Colposcopic Classification (2011, supplemented in 2017).

### 3.9. RNA Isolation and Analysis

Total RNA was isolated from endometrial samples obtained by pipelle biopsy on days 7–10 of the menstrual cycle from women with RPL and healthy women one month after pregnancy termination. Histological blocks were processed, and RNA was extracted using the guanidine–thiocyanate–phenol–chloroform method (Eurogen, Moscow, Russia). RNA quality and concentration were adequate for quantitative measurements, with results analyzed by real-time PCR. These methodologies ensured a comprehensive analysis of the molecular biological changes in the endocervix and endometrium of women with unexplained RPL.

### 3.10. Evaluation of miRNA Expression: RT-PCR

To detect mature miRNAs, the Stem-loop RT-qPCR method described by Kramer et al. [39] was used. The isolated total RNA was used in a reverse transcription reaction with Stem-loop RT primers specific for each of the detected miRNAs. Then, the obtained complementary DNA (cDNA) was detected using RT-PCR using the intercalating dye SYBR Green I. The design of specific oligonucleotide primers was carried out using the algorithm of Kramer et al. [39] and the primers are given in Table 10.

For each miRNA, a reverse transcription reaction was performed separately on 3 μL of isolated total RNA in 1 repeat using the MMLV RT kit (Eurogen, Russia), according to the instructions, using 100 U of MMLV reverse transcriptase and a final concentration of 1 μM primer for RT. The reaction was carried out for 60 min at 37 °C, and then the reverse transcriptase was inactivated for 10 min at 70 °C. At the same time, a completely duplicate reaction in all parameters and reagents without reverse transcriptase (replaced with the appropriate volume of water) was carried out in a separate test tube for the control without transcriptase. Changes in relative miRNA expression were assessed by real-time PCR with the intercalating dye SYBR Green I using the 5X qPCRmix-HS SYBR master mix (Eurogen, Moscow, Russia), according to the instructions. The amplification was performed at a volume of 25 μL at a concentration of 200 nM of direct and reverse primers. Quantitative real-time PCR for each sample was performed in triplicate for each sample and each determined miRNA, with a control without transcriptase. The resulting mixtures were incubated in a CFX 96 amplifier (Bio-Rad, Hercules, CA, USA) according to the following program: 5 min at 95 °C for 40 cycles accompanied by denaturation at 95 °C for 10 sec, whereas annealing was carried out at 59 °C for 20 s and elongation at 72 °C for 20 s. After PCR, melting curves were ascertained to assess the specificity of the PCR product and the absence of primer dimers (one peak on the melting curve). The results corresponding to threshold cycle (Ct) values >40 were considered negative, amplification curves in controls without reverse transcription were absent, and melting curves corresponded to a specific PCR product.

All oligonucleotides were synthesized by Eurogen (Moscow, Russia). The efficiency of reverse transcription was estimated by the values of threshold cycles (Ct) when analysing serial dilutions of the analyzed matrix samples obtained after reverse transcription. A graph of the dependence of Ct on the logarithm of the initial amount of matrix (log10) was constructed. To determine the slope of the curve *m*, the linear regression method is used, which fits the linear equation Y = mX + b to the data, where Y is the Ct value, and X is the log10 of the initial amount of matrix. The slope *m* of this line is then used to calculate the efficiency (E) of PCR using the formula E = (10^−1^/m^−1^). This calculation allows us to estimate the degree of DNA doubling in each PCR cycle, reflecting the amplification efficiency, and is used to calculate relative expression. The expression analysis of hsa-miR-16-5p, which is stably expressed in many tissue types, was used as a reference RNA [40]. The relative expression (RE) was calculated using the formula RE = E^−ΔΔCt^ (E is the amplification efficiency, E = 2.0). The results were normalized using the reference loci and the expression level of miRNA in the control group samples sequentially according to the scheme below.

The 2^−ΔΔCt^ method was implicated for examining relative expression of miRNA genes. First, the difference in threshold cycles (Ct) for the target and reference miRNAs of each sample is calculated (ΔCt = Ct target − Ct reference). Then, relative to the control sample, ΔΔCt is calculated for each sample (ΔΔCt = ΔCt sample − ΔCt control). The relative expression (RE) is defined as RE = (E + 1^−ΔΔCt^, where E is the amplification efficiency.

### 3.11. Statistical Analysis

The StatSoft Statistica v10 Advanced software package (Statsoft Ins., Tulsa, OK, USA; license No. AXAR507G794202FA-B) was used for statistical data processing. The normality of data distribution was examined with the aid of Shapiro–Wilk test. In case of normal distribution, data described the arithmetic mean and standard deviation (M ± σ) or the median and 25th and 75th percentiles (Me [Q1; Q3]) for a distribution that differed from normal. When comparing categorical indicators, the two-sided Fisher test was used. The degree of microRNA expression in the groups was ascertained by the Wilcoxon–Mann–Whitney test. Differences were considered statistically significant at *p* ≤ 0.05.

## 4. Discussion

The qualitative and quantitative composition of the cervicovaginal microbiota of women with RPL of unknown genesis was distinguished by a higher frequency of BV and urogenital infections, especially genital herpes. In the group with RPL, in contrast to healthy pregnant women, more than half of the patients had an abnormal cytological picture (ASC-US and LSIL). The vaginal microbiota of women with RPL was distinguished by the predominance of G. vaginalis and the infectious potential of the cervical microenvironment was determined by a high diagnostic titer of Ureaplasma urealyticum (105) and infection with HPV HR in 47.4% of women with RPL. The prevalence of STIs (*Mycoplasma genitalium*, HSV2) in the sample with RPL indicates the need for screening and treatment before planning a pregnancy. Apparently, the diversity of the vaginal microbiota of women with RPL (BV and deficiency of *Lactobacillus* spp.) determines a favorable background for the life cycle of the virus, which is most dissociative in abnormal cytograms. Our data confirm the relationship between cervicovaginal microbiome disorders and HPV infection, HPV HR and BV, precancerous changes and cervical cancer [41,42,43]. HPV infection also contributes to the imbalance of the cervical and vaginal microbiota, affecting the expression of host defense peptides of the mucous membrane. HPV oncoprotein E7 significantly inhibits host defense peptide expression, such as HβD1, 2, 4, HD-5/6, SLPI, S100A7 and elafin, which could interact with NF-κB as well as Wnt/β-catenin signaling [44]. The coexpression of p16 and the cell cycle progression biomarker Ki-67 in a single cell, detected in 52.3% of women with RPL, allows us to identify epithelial cells transformed by HPV (47.3%). Aberrant expression indicates unregulated proliferation of endocervical cells and the risk of precancerous and cancerous changes [21,22].

The results obtained allow us to state that the formation of a pathological microbial community is associated with dystrophic changes in the cervical epithelium, disruption of intercellular contacts, and inflammatory infiltration of the stroma. BV-associated bacteria are involved in oxidative stress reactions when co-cultivated with a three-dimensional model of the cervical epithelium, which affects the integrity of the epithelial barrier [45]. The analysis of the cervicovaginal composition of women with RPL from the standpoint of searching for conditions for the implementation of pathogenic properties of microorganisms unfavorable for prolongation of pregnancy confirms the involvement of local immune mechanisms. Violation of the colonization resistance of the cervicovaginal biotope with the transformation of the immunological environment is most vivid in cases of HPV HR infection. A high level of Th17/Th1 lymphocytes in the endocervix with decreased expression of protective CD4+ T lymphocytes in the group of women with RPL may indicate immunoregulatory disorders of tolerance to a semi-allogenic fetus. Changes in the lymphocyte balance with the dominance of proinflammatory Th17/Th1 in the endocervix of women with pregnancy losses allow us to discuss the relationship between immune cells and specific microbiota modulating local immune responses. The predominance of Th17 and IL-17 expression in the endocervix during HPV persistence is associated with an enhanced cervical immune response and progression of cervical lesions [46].

The reduced expression of mRNA of the gene encoding PGR-A in endocervical cells of women with RPL indicates a disruption of progesterone-mediated responses, both through a direct effect on decidual immune cells and by stimulating Treg differentiation at the mother–fetus interface. An anti-inflammatory effect of this hormone on T-cell-mediated inflammatory processes at the mother–fetus interface is suggested [47]. In contrast, the increased expression of IL-17 is involved in fetal rejection by the maternal immune system and implantation failure [48]. For a pregnancy to proceed successfully, maternal tissues undergo significant structural and physiological modifications throughout various stages. Initially, embryo implantation requires the remodeling of the endometrial lining to support the attachment and growth of the blastocyst. Following implantation, placentation involves the development of a complex interface between maternal and fetal tissues, facilitating nutrient and gas exchange. Angiogenesis, or blood vessel formation, further enhances the vascular supply to meet the demands of the growing fetus. Hemostasis must be carefully regulated to prevent both excessive clotting and hemorrhage, ensuring stable blood flow to the placenta. Additionally, immune tolerance is essential; the maternal immune system must adapt to accept the genetically distinct fetus, involving shifts in immune cell populations and cytokine profiles that suppress potential rejection responses while still protecting against infections. Each of these processes is tightly coordinated to ensure an optimal environment for fetal development [49]. Vaginal *Lactobacillus* suppresses the proinflammatory response of epithelial or immune cells and promotes the polarization of M2 macrophages, which promotes the differentiation of CD4+ T cells into immunoregulatory Treg cells [50]. The prevalence of abnormal cervicovaginal microbiota, the highest dysbiotic disorders and intraepithelial lesions of the cervix in HPV-positive pregnant women with RPL, suggests a continuum of changes: vaginal biotope–cervical epithelium–embryo-trophoblast interactions.

The immunocytochemical presentation of increased proliferative potential of endocervical cells (hyperexpression of p16/Ki-67, apoptosis regulator BCL-2, mRNA-34 and -145) with abnormal cytograms (ASC-US, LSIL) confirms the commonality of molecular biological mechanisms of RPL and HPV-associated lesions of the cervix, accompanied by damage to the receptor apparatus (decreased expression of mRNA of the gene encoding PGR-A) and the development of progesterone resistance. We believe that the mechanism of RPL of unknown genesis is a violation of the uterine–embryotrophoblast interaction caused by the excessive proliferation of endocervical cells and the development of progesterone resistance. The persistence of HR HPV probably triggers a cascade of molecular and cellular “breakdowns” in the endocervical receptor apparatus with the disruption of intra- and intercellular signal transmission.

It is reported that miR-145 can play an important role in regulating tumor cell progression, migration, invasion, and apoptosis of some types of cancer by affecting c-Myc, Mucin-1, p70S6 kinase, and insulin receptor substrate [51,52,53]. The expression profile of miR-34a, which is involved in proliferation processes, is considered promising for early diagnosis, classification, or prognosis of neoplastic processes in various biological environments of the body [54,55]. The dysfunction of miR-34a is associated with a neoplastic mechanism that is activated by oncoprotein E6 and p53 during HPV persistence [56]. The decreased expression of miR-145 and miR-34a in the endocervix of patients with ASC-US and LSIL, mainly in the group with RPL, is the result of disrupted cellular proliferation. Our data on the low expression of miR-145 and miR-34a in the endometrial samples of women with RPL compared to the group with physiological pregnancy contradict the results of the relationship between recurrent pregnancy losses exclusively with elevated miR levels [57,58]. The multidirectional expression pattern of anti-oncogenic signaling molecules may vary depending on the presence of HR HPV in the endocervix. Changes in the endometrium leading to reproductive failures are due to decreased expression of miR against the background of persistent HPV infection long before pathomorphological changes in the cells of the cervix. The decreased expression of miR-145 and miR-34a not only in the endocervix but also in the endometrium of women with RPL allows us to express the concept of immunoregulatory damage of impaired trophoblast invasion, remodeling of spiral arteries and development of the placental bed. The violation of hormonal-mediated effects of PlGF, realized through fms-like tyrosine kinase-1, is due to decreased expression of PGR in the endocervical epithelium, one of the key links in the pathogenesis of RPL of unknown genesis.

The mechanisms of RPL with increased expression of miR-145 and miR-34a in the group without HR HPV infection may be due to changes in the activity of receptors tyrosine kinase (RTKN), estradiol (ER-α), insulin-like growth factor 1 (IGF-1R) and other signaling factors [59]. Data on the implementation of the effects of increased expression of miR-34a through the Wnt/β-catenin signaling pathway that could regulate the proliferation and invasion of trophoblast cells by inactivation in vitro are also presented [60].

The results of our study suggest that low levels of miR-34a and miR-145 expression not only in the endocervix but also in the endometrium are associated with impairment in regulating cell cycle and apoptosis and the development of endometrial dysfunction. The ambiguity of the data on the nature of changes in miR expression—from decreased to hyperexpression—is due to the complexity of the key cascades of intracellular signaling that regulate the process of implantation, trophoblast cell invasion and the development of gestational complications. It is necessary to point out the lack of complete information on potential miR target genes and the proteins they encode in the endometrium of women with RPL. The revealed decrease in miR expression in the endocervix, especially with the persistence of HR HPV, suggests an adverse effect of the hyperimmune response on the processes of cellular metabolism, implantation and trophoblast development. The idea of the role of immune factors in the genesis of RPL is consistent with the data on the involvement of miR in the modulation of the expression of genes associated with the synthesis of inflammatory cytokines, chemokines, and other molecules that are potentially involved in the development of the placenta [61]. The violation of the local immune status (prevalence of proinflammatory Th17/Th1 T-lymphocytes against the background of a low level of regulatory CD4+ Tregs) in women with RPL was associated with a decrease in the functional activity of miRs regulating target genes that control the interaction of the embryo–placenta interface. The data obtained by us suggest that potential regulators of cellular processes, i.e., the immune response, viral infection, the change in expression of which affects the ability to implant and carry a pregnancy, also include their interaction with target genes.

## 5. Conclusions

Our findings elucidate the intricate interplay between cervicovaginal microbiota characteristics, local immune status, and the hormonal receptor apparatus of the cervix in the context of recurrent pregnancy loss (RPL). The established cause-and-effect relationships between molecular and cellular alterations in the endocervix and recurrent miscarriage underscore new avenues in the pathophysiological understanding of reproductive losses. Notably, our data reveal that dysregulation of signaling molecules in the endometrium further contributes to this complex network of interactions.

The integration of advanced methodologies such as liquid cytology, flow cytometry, and immunocytochemical techniques has proven instrumental in diagnosing features associated with RPL risk. Furthermore, our observations indicate that oncogenic human papillomavirus (HPV) infections in nearly half of the studied pregnant women are correlated with significant alterations in cervicovaginal microbiota, cellular composition of the endocervix, and disruptions in local immune balance.

The notable disturbances in the expression of miR-34a and miR-145 in the endocervical and endometrial samples from women experiencing RPL highlight the potential of these microRNAs as biomarkers for miscarriage risk. These findings open new frontiers for investigating the complex molecular mechanisms and signaling pathways underlying epigenetic control of embryo development and the mother–fetus interface. Additionally, exploring the mechanistic roles of miR-34a and miR-145 in regulating endocervical and endometrial functions will enhance our understanding of the molecular underpinnings of RPL. Ultimately, establishing a comprehensive biomarker panel could facilitate personalized interventions aimed at improving reproductive outcomes for women at risk of recurrent pregnancy loss.

The revealed association of disorders of the cervicovaginal microbiota and the immune system of the endocervix, the influence of which on the interaction of the embryo and the endometrium is proved by the violation of microRNA expression in the endometrium of women with RPL of unknown genesis, requires the correction of dysbiotic and inflammatory conditions of the lower genital tract, antiviral therapy and support with gestagens.

## Figures and Tables

**Table 1:** Clinical parameters of women with RPL and healthy pregnant women.

Variables	RPL (n = 239)	Healthy Women (n = 118)	*p*
Age (average value ± SD)	32.7 ± 3.2	33.9 ± 4.4	>0.05
Body mass index (BMI)	26.8 ± 5.4	28.4 ± 3.5	>0.05
BMI ≥ 30 kg/m^2^	46 (19.2%)	17 (14.4%)	>0.05
Parity			
First-time mothers	142 (59.4%)	75 (63.6%)	>0.05
History of diseases			
Hypertension	43 (18.0%)	11 (9.3%)	>0.05
Anemia	54 (22.6%)	20 (16.9%)	>0.05
Diabetes mellitus	15 (6.3%)	6 (5.1%)	>0.05
Urinary tract infections	21 (8.8%)	14 (11.9%)	>0.05

**Table 2:** Results pertinent to vaginal smears of women with RPL and healthy pregnant women.

Parameters	RPL (n = 239)	Control (n = 118)	*p* (χ^2^)
Normocenosis	44 (18.4%)	68 (57.6%)	0.001 (56.1)
Non-specific vaginitis	65 (27.2%)	25 (21.2%)	>0.05
Bacterial vaginosis (BV)	81 (33.9%)	15 (12.7%)	0.001 (18.0)
Candida vaginitis	49 (20.5%)	10 (8.5%)	0.04 (8.3)

**Table 3:** Prevalence of microorganisms isolated in vaginal discharge culture in women with RPL and healthy pregnant women.

	RPL	Control Group	*p*	RPL	Control Group	*p*
	Overall			Opportunistic Microorganisms in High Diagnostic Titer (>10^4^)		
*Enterococcus* spp.	90 (43.9%)	10(8.5%)	<0.001 45.5	82 (34.3%)	6 (5.1%)	<0.001 36.3
*Streptococcus agalactiae*	90 (37.7%)	8 (6.8%)	<0.001 37.8	75 (31.4%)	6 (5.1%)	<0.001 31.1
*Streptococcus* spp.	15 (6.3%)	9 (7.6%)	>0.05	7 (2.9%)	5 (4.2%)	>0.05
*Corynebacterium* spp.	15 (6.3%)	5 (4.2%)	>0.05	15 (6.3%)	2 (1.7%)	>0.05
*Klebsiella* spp.	13 (5.4%)	0 (0.0%)	>0.05	7 (2.9%)	0 (0.0%)	>0.05
*Escherichia coli*	52 (21.8%)	9 (7.6%)	<0.001 11.1	45 (18.8%)	3 (2.5%)	<0.001 18.0
*Staphylococcus* spp.	30 (12.6%)	12(10.2%)	>0.05	7 (2.9%)	5 (4.2%)	>0.05
*Staphylococcus epidermidis*	22 (9.2%)	6 (5.1%)	>0.05	15 (6.3%)	4 (3.4%)	>0.05
*Actinomyces* spp.	22 (9.2%)	15 (12.7%)	>0.05	7 (2.9%)	5 (4.2%)	>0.05
*Corynebacterium* spp.	19 (7.9%)	6 (5.1%)	>0.05	3 (1.3%)	3 (2.5%)	>0.05
*Bacillus* spp.	8 (3.3%)	4 (3.4%)	>0.05	0 (0.0%)	4 (3.4%)	>0.05

**Table 4:** An analysis of the occurrence of various types of HPV, in combination with a violation of the vaginal biotope and the magnitude of the viral load in groups with recurrent pregnancy loss (RPL) and healthy pregnant women with HPV.

Parameters	RPL (n = 113)	Control (n = 9)	*p* (χ^2^)
HPV HR	68 (60.2%)	2 (22.2%)	0.001 (16.8)
HPV 16 type	43 (31.1%)	1 (11.1%)	0.005 (8.1)
HPV + bacterial vaginosis	85 (75.2%)	1 (11.1%)	0.001 (35.1)
Two or more HPV types	68 (60.2%)	0 (0.0%)	-

**Table 5:** Results of liquid cytology of exo- and endocervix scrapings of the examined women.

Parameters	RPL (n = 239)	Control (n = 118)	*p* (χ^2^)
NILM (negative for intraepithelial lesion or malignancy)	150 (62.8%)	95 (80.5%)	0.001 (11.6)
ASC-US (atypical squamous cells of undetermined significance)	47 (19.7%)	12 (10.2%)	0.02 (5.2)
LSIL (low-grade intraepitelial lesion)	42 (17.6%)	11(9.3%)	0.04 (4.2)

**Table 6:** Frequency of abnormal expression of markers p16, Ki-67, BCL-2 and PGR-A in the endocervix of women with RPL and healthy pregnant women.

Markers	RPL (n = 44)	Control (n = 222)	*p* (χ^2^)
p16	23 (52.3%)	4 (18.2%)	0.005 (7.9)
Ki-67	23 (52.3%)	4 (18.2%)	0.005 (7.9)
BCL-2	24 (54.5%)	3 (13.6%)	0.002 (10.1)
PGR-A	22 (50.0%)	3 (13.6%)	0.005 (8.2)

**Table 7:** Features of immunocompetent cells (Th17/Th1 ratio and CD4+ Tregs) in the endocervix of women with RPL and healthy pregnant women.

Parameters	RPL (n = 44)	Control (n = 22)	*p* (χ^2^)
Th17/Th1	22 (50.0%)	3 (13.6%)	0.005 (8.2)
CD4+ Treg	10 (22.7%)	16 (72.7%)	0.001 (15.3)

**Table 8:** The disruption (decrease) of miRNA expression in women with RPL and healthy pregnant women.

Parameters	RPL (n = 44)	Control (n = 22)	*p* (χ^2^)
miR-145	24 (54.5%)	3 (13.6%)	0.002 (10.1)
miR-34a	27 (61.4%)	3 (13.6%)	0.001 (13.5)

**Table 9:** Expression of miR-145 and miR-34a in the endometrium of women with RPL and healthy pregnant women.

Parameters	RPL (n = 24/27)	Control (n = 4)	*p*
miR-145 (HPV HR +) (n = 16)	0.8 (0.7; 0.9)	1.1 (1.0;1.2)	<0.05
miR-145(HPV HR −) (n = 8)	1.6 (1.5; 1.7)		<0.05
miR-34a (HPV HR +) (n = 16)	0.6 (0.5; 0.7)	1.1 (1.0;1.2)	<0.05
miR-34a (HPV HR −) (n = 11)	1.85 (1.7; 2.0)		<0.05

**Table 10:** The following primers were used for reverse transcription PCR.

miR	Primer for Reverse Transcription	Direct PCR Primer	Reverse PCR Primer
hsa-miR-145-5p	5′GTCGTATCCAGTGCAGGGTCCGAGGTATTCGCACTGGATACGACAGGGA3′3′	5′AACAAGGTCCAGTTTTCCCAG3′	5′GTCGTATCCAGTGCAGGGT3′
hsa-miR-34a-5p	5′GTCGTATCCAGTGCAGGGTCCGAGGTATTCGCACTGGATACGACACAACC3′	5′AACAGTGTGGCAGTGTCTTAG 3′	5′GTCGTATCCAGTGCAGGGT3′
hsa-miR-16-5p	5′GTCGTATCCAGTGCAGGGTCCGAGGTATTCGCACTGGATACGACCGCCAA3′	5′AACAGTGTAGCAGCACGTAAA3′	5′GTCGTATCCAGTGCAGGGT3′

## Data Availability

Data are contained within the article.
